# Can China’s Carbon Emissions Trading System Achieve the Synergistic Effect of Carbon Reduction and Pollution Control?

**DOI:** 10.3390/ijerph19158932

**Published:** 2022-07-22

**Authors:** Li Chen, Di Wang, Ruyi Shi

**Affiliations:** 1 School of Economics and Management, China University of Mining and Technology, Xuzhou 221116, China; 15866736650@163.com; 2 School of Economics, Shandong Women’s University, Jinan 250300, China; 3 Think Tank of Carbon Neutral and Energy Strategy, China University of Mining and Technology, Xuzhou 221116, China; 4 School of Public Policy and Management, China University of Mining and Technology, Xuzhou 221116, China; 5 School of Safety Engineering, China University of Mining and Technology, Xuzhou 221116, China

**Keywords:** carbon emissions trading system, time-varying difference-in-differences model, synergistic emission reduction, air pollution

## Abstract

Achieving synergistic governance of air pollution treatment and greenhouse gas emission reduction is the way for the Chinese government to achieve green transformational development. Against this background, this paper takes the implementation of the carbon emissions trading system (ETS) as the breakthrough point, using the time-varying difference-in-differences (DID) model to explore the synergistic emission reduction effect of ETS on air pollution and carbon emissions and its mechanism. The results indicate that the implementation of ETS not only significantly reduces CO_2_ emissions but also synergistically achieves the reduction of air pollutants, and the synergistic emission reduction effect is mainly achieved through the synergistic reduction of SO_2_. Moreover, the emission reduction effect of ETS has economic and regional heterogeneity. On the one hand, the ETS has a more prominent carbon reduction effect in less developed provinces and cities and has a significant synergistic emission reduction effect on SO_2_ and PM_2.5_; on the other hand, the carbon emission reduction effect of ETS is more potent in Beijing, Hubei, and Shanghai, followed by Tianjin and Chongqing, and the weakest in Guangdong. In addition, through the analysis of the mediating effect, this paper finds that reducing energy consumption, optimizing the energy structure, and improving energy efficiency are effective ways for ETS to achieve synergistic emission reduction. This study provides valuable policy enlightenment for promoting the synergistic governance of pollution and carbon reduction.

## 1. Introduction

In recent years, global warming and the frequent occurrence of extreme weather have seriously affected human life, while air pollution has also become increasingly prominent. Greenhouse gas emissions and air pollution are global environmental problems that affect the sustainable development of human beings. However, the synergistic management of these two problems has not achieved the expected results, and has raised the total cost to society [1,2]. As the world’s largest carbon emitter, China faces many challenges to the synergistic development of its regional economy, energy resources, and ecological environment, especially greenhouse gas emissions with CO_2_ as the main body; cross-regional and compound air pollution has become more and more serious [3,4]. As the world’s largest developing country and the largest carbon dioxide emitter, China has actively implemented a national strategy to address climate change and has made crucial carbon reduction commitments to the international community to reach peak carbon emissions by 2030 and achieve carbon neutrality by 2060 [5]. Meanwhile, to reduce pollution and carbon emissions, the Chinese government has also clearly proposed to establish the concept that lucid waters and lush mountains are invaluable assets, and to fight a tough battle for pollution prevention and control. The Fifth Plenary Session of the 19th CPC Central Committee and the Central Economic Work Conference have put forward precise requirements for achieving the synergistic effect of pollution reduction and carbon reduction [6]. During the “14th Five-Year Plan” period, China’s ecological and environmental protection will enter a new stage of synergistic governance of pollution reduction and carbon reduction, and how to achieve these goals has become the focus of social attention [7]. China’s greenhouse gas emission reduction efforts were carried out late, and for a long time, China did not carry out coordinated treatment of greenhouse gases and pollutants. In contrast, developed countries, benefiting from economic and technological advantages, are leading the way in pollutant control and GHG emission reduction.

The ETS is considered an effective means of reducing global greenhouse gas emissions and coping with climate change because of its effectiveness, flexibility, and cost savings [8,9]. In 2011, the National Development and Reform Commission (NDRC) officially approved Beijing, Tianjin, Shanghai, Chongqing, Hubei, Guangdong, and Shenzhen to launch a carbon emissions trading pilot. Then, the carbon trading markets of seven provinces and cities were opened from June 2013 to June 2014. In December 2016, Fujian Province launched the carbon trading market as China’s eighth carbon trading pilot. The power generation industry was the first to be included in the national carbon trading market in 2021. According to the Chinese government’s work report for 2021, the government will implement the expected GHG emission reduction targets for 2030 during the 14th Five-Year Plan period, achieving an 18% and 13.5% reduction in CO_2_ emissions and energy consumption per unit of GDP, respectively, and accelerating the construction of a national carbon emissions trading market [5]. The carbon emissions trading market will gradually become an essential means of coping with climate change and preventing environmental pollution in China [10]. Some questions need to be verified, whether ETS has a significant synergistic emission reduction effect, and what mechanism is used by ETS to play its synergistic emission reduction role? This paper explores the above issues based on systematic and rigorous empirical research. The quantitative research on the synergistic emission reduction effect of the ETS has solid practical significance for China to comprehensively promote the response to climate change and ecological environment protection.

Scholars have conducted numerous studies on environmental issues from different perspectives. Many studies have discussed the relationship between environmental pollution and economic growth, but no consensus has been reached [11]. Schmalensee et al. studied the relationship between per capita income and CO_2_ emissions in many countries over 40 years using a piecewise linear function and confirmed the existence of the EKC curve [12]. Lantz and Feng found no correlation between CO_2_ emissions and per capita income [13]. Subsequent scholars studied the relationship between SO_2_, NO_x_, and per capita income, and the results were quite different [14,15]. In addition, the issue of free trade and environmental pollution has also attracted the attention of scholars. Walter and Ugelow first proposed the “pollution paradise” hypothesis, arguing that high pollution industries in developed countries would shift to developing countries with low environmental standards [16]. That is, FDI exacerbates environmental pollution problems in developing countries [17,18]. However, some scholars hold a different view that restricting FDI cannot reduce carbon emissions but will instead have negative impacts [19]. The impact of FDI on the local environment is complex: on the one hand, FDI can promote pollution reduction through technology spillover, and on the other hand, it may also aggravate local environmental pollution through the introduction of energy-intensive and high-polluting enterprises, which mainly depends on the level of local environmental regulation. As a market-based environmental regulation instrument, can ETS achieve the synergistic reduction of carbon emissions and air pollutants in regions with different levels of foreign capital utilization by promoting technological progress or energy structure optimization? This question remains to be further tested. At present, the research on ETS is mainly based on developed countries. According to the research data from the World Bank, the implementation of carbon emissions trading reduced carbon emissions by an average of 40 million to 100 million tons per year from 2005 to 2007 [20]. Streimikiene et al. studied the changes in carbon emissions of the Baltic States from 2005 to 2007 and concluded that the European Union’s Greenhouse Gas Emissions Trading System (GHG) had not achieved the expected results [21]. Borghesi et al. found that the European carbon emissions trading system significantly reduced the carbon emissions of the Italian manufacturing sector [22]. Naegele and Zaklan used the EU Greenhouse Gas Emissions Trading System (EU-ETS) as the research object and found no carbon leakage in European manufacturing [23].

The studies on China’s ETS mainly focus on two significant aspects. One is to use multiple models to scenario simulate the economic impact or environmental impact of ETS [24,25]. For example, Liu et al. analyzed the impact of ETS in Hubei province using a CGE model, and the results showed that the ETS significantly reduced carbon emissions, while their negative impact on the economy was relatively negligible [26]. Tang et al. constructed a bottom-up dynamic carbon trading mechanism simulation model based on a multi-agent model to analyze the impact of carbon trading mechanisms on the economy while reducing carbon emissions [27]. The other is to evaluate the effect of ETS by constructing quasi-natural experiments to reduce estimation bias using DID model. In the existing literature, the DID model is often used to verify and analyze the impact of the ETS on the sustainable reduction of CO_2_ emissions [28,29,30]. Zhang et al. analyzed the impact of ETS on industrial carbon emission reduction and found that ETS can reduce industrial carbon emissions and carbon intensity by 10.1% and 0.78%, respectively, while industrial energy efficiency plays a crucial role in emission reduction [31]. Yi et al. found that ETS did not affect carbon emission reduction in all pilot regions [32]. Wang et al. [33] and Zhang et al. [34] analyzed the impact of ETS on carbon emissions and economic growth using panel data from 30 provinces and cities in China and found that ETS can achieve both environmental and economic benefits. Liu et al. [35] analyzed the emission reduction effect of ETS on PM_2.5_ by using the monthly data of 297 cities.

In summary, there is no consistent conclusion on the effectiveness of ETS, and the shortcomings of existing research are mainly reflected in the following aspects. First, most empirical analysis literature only focuses on the emission reduction effect of ETS. It lacks a systematic and integrated perspective on the problems of CO_2_ emission reduction and air pollution control, which separates the two homologous issues of CO_2_ emission reduction and air pollution control from each other, with insufficient consideration of the synergistic governance mechanism and compatible policy system for pollution reduction and carbon reduction. Although some scholars have evaluated the synergistic effect of ETS, they did not analyze the synergistic effect of ETS on air pollutant reduction from the perspective of synergistic emission reduction, ignoring the spillover effect of ETS on air pollutant reduction, making it challenging to achieve synergistic management of CO_2_ and air pollutants effectively [36]. Second, although the simulation methods have been widely used, they have too many assumptions, complicated internal design, and difficulty in tracing their mechanism. The choice of model parameters affects the conclusions, making it challenging to reflect the real effects of ETS fully. Third, the DID model is favored by many scholars as an effective method for policy evaluation. However, part of the literature ignores the premise assumptions of the method and does not conduct parallel trend tests, which cannot guarantee that the experimental and control groups have the same trend before policy implementation, which may lead to biased estimation results and cannot accurately determine the carbon reduction effect of ETS [30].

The marginal contributions of this paper are as follows. First, CO_2_ and air pollutants are included in a unified research framework to examine the emission reduction effectiveness of ETS comprehensively. The time-varying DID model is used to empirically analyze the synergistic emission reduction effect of ETS, which is conducive to the synergistic control of carbon dioxide and air pollutants [8]. Second, this article uses the mediation model to analyze the mechanism of synergistic emission reduction of the ETS and finds that ETS can achieve synergistic emission reduction by optimizing the energy consumption structure and promoting technological progress. Third, the conclusions of this paper can provide empirical support and policy recommendations for China to improve the carbon emissions trading market mechanism and actively and steadily promote the further construction of the carbon emissions trading market.

The following structure of this paper is arranged as follows: Section 2 is a theoretical analysis and research hypotheses; Section 3 is the methodology and data; Section 4 reports the empirical results and hypothesis testing; Section 5 is the analysis and discussion; Section 6 puts forward conclusions; Section 7 puts forward policy recommendations and prospects.

## 2. Theoretical Analysis and Hypothesis

Carbon trading is a market mechanism that trades carbon dioxide emission rights as a commodity. Its essence is to use the market economy to achieve environmental protection, compensate for the limitations of command-and-control ecological policies, and avoid government failure. The carbon trading mechanism is derived from emissions trading, and the theoretical basis of emissions trading is the Coase theorem. Carbon emissions have externalities because they belong to the category of public goods. Coase [37] believes that the root of market inability lies in the failure of property rights and that external costs can be internalized through a clear definition of property rights. The carbon trading system is to make carbon emissions become non-public goods. Carbon emissions trading means that when the national total carbon emissions target is set, the government decomposes the total carbon emissions target layer by layer according to specific rules and determines market participants’ initial carbon emission quotas. The government encourages enterprises with low emission reduction costs to reduce excess emissions and sells the surplus carbon emission quotas to enterprises with high emission reduction costs and unable to complete the emission reduction target through carbon trading so that under the action of the market mechanism, it can control the total amount of CO_2_ emissions at low costs and high efficiency. Because the emissions of CO_2_, SO_2_, PM_2.5_, and other atmospheric pollutants are primarily due to the burning of fossil fuels with the same root and origin [38], the implementation of ETS can also synergistically reduce the emissions of atmospheric pollutants. Given this, this paper puts forward:

**Hypothesis** **1.** *Carbon emissions trading system can reduce CO_2_ emissions and bring about the synergetic reduction of air pollutants.*

However, how does carbon emissions trading achieve synergistic emission reduction? The implementation of ETS internalizes the externalities of carbon emissions into the production costs of enterprises, which brings cost pressure. Enterprises can choose to optimize the energy structure and use clean energy to reduce carbon emissions or purchase quotas from the carbon trading market to compensate for the emissions gap. Enterprises will weigh the price of carbon trading against the marginal cost of emission reduction and ultimately achieve the emission reduction targets set by the government at a lower cost. In the long run, to reduce costs, enterprises will adjust the production mode and choose clean or “zero-carbon” energy to optimize the energy structure and reduce carbon emissions.

The Porter hypothesis suggests that appropriate environmental regulation can promote technological progress, which in turn leads to productivity gains, make up for the cost of pollution control, and improve firm competitiveness [39]. Under the theoretical framework of Porter’s hypothesis, the implementation of ETS enables enterprises to remeasure the cost of purchasing carbon quotas and the cost of improving carbon reduction technology, forcing enterprises to carry out technological research and development and promote technological progress [40]. Technological progress can apply high-efficiency production equipment and processes to improve energy efficiency, reducing carbon emissions. Enterprises can also sell their rich carbon quotas in the carbon trading market and then profit by focusing on low-carbon technology research and development to make up for the cost of pollution control, forming a virtuous circle [41].

**Figure 1:**
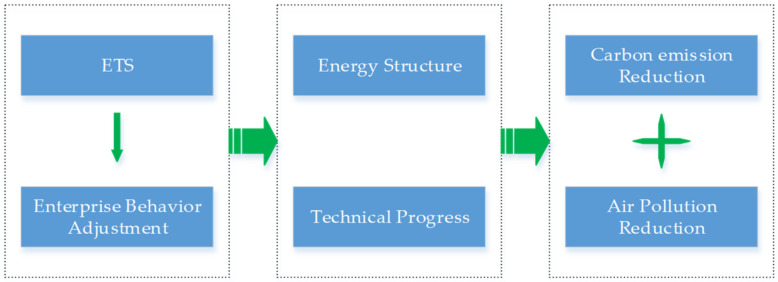
Synergistic emission reduction mechanism of ETS.

Under the action of ETS, enterprises will choose to optimize energy structures and promote technological progress to reduce carbon emissions. On the one hand, enterprises will reduce coal consumption, and coal-burning will produce harmful gases such as CO_2_, SO_2_, and PM_2.5_, so it will bring carbon emission reduction when reducing coal consumption, which can also synergistically reduce the emission of air pollutants. On the other hand, in the long run, enterprises will promote technological progress. Technological progress can improve energy utilization efficiency and achieve carbon emission reduction while reducing pollutant emissions, reflecting the spillover effect of ETS. Its transmission mechanism is shown in Figure 1.

Given the above analysis, this paper puts forward the following hypotheses:

**Hypothesis** **2.** *Carbon emissions trading system can synergistically reduce the emissions of CO_2_ and air pollutants through the effect of optimizing energy structure*.

**Hypothesis** **3.** *Carbon emissions trading system can synergistically reduce the emissions of CO* *_2_ and air pollutants through the effect of technological progress.*

## 3. Methodology and Data

### 3.1. Methodology

In 2011, with the approval of the National Development and Reform Commission, carbon emissions trading pilots were carried out in Beijing, Tianjin, Shanghai, Chongqing, Hubei, Guangdong, and Shenzhen. Because Shenzhen belongs to Guangdong Province, and other pilot areas are provinces or autonomous regions, this paper classifies Shenzhen into Guangdong Province for analysis. From June 2013 to June 2014, carbon emissions trading was launched in the above pilot provinces and cities, so this paper takes 2014 as the year when the ETS affected the above areas. In December 2016, Fujian Province launched the carbon emissions trading market, and this paper regards 2017 as the year when Fujian Province began to be affected by it. The problem to be investigated in this paper is whether the implementation of ETS can be synergistically effective in reducing regional CO_2_ and air pollutant emissions. To solve the endogeneity issue, this paper regards the ETS as a quasi-natural experiment, taking the seven provinces and cities mentioned above as the experimental group and other non-pilot provinces and cities as the control group. Due to the inconsistent time of starting ETS in the seven pilot areas [6], this paper applies a time-varying DID model to test the impact of ETS on carbon dioxide and air pollutant emissions. Based on the above analysis, this paper constructs the following econometric model concerning the research ideas of Cheng et al. [42].

(1) $$Emission_{it}=\beta_{0}+\beta_{1}DID_{it}+\beta_{2}X_{it}+\alpha_{i}+\gamma_{t}+\varepsilon_{it}$$

where *i* represents the region, and *t* represents the year. $DID_{it}$ is the key explanatory variable, that is, a time-varying DID variable ($DID_{it}=treat_{i}\times post_{it}$), $treat_{i}$ is a regional dummy variable indicating whether city *i* implements the ETS, and $post_{it}$ is a time dummy variable indicating whether the ETS is implemented in year *t*. If the coefficient *β*_1_ is significantly negative, it indicates that the ETS is effective in synergistically reducing CO_2_ and air pollution emissions. The explained variable “*Emission*” includes emissions of CO_2_, SO_2_, and PM_2.5_. $X_{it}$ represents a series of control variables. $\alpha_{i}$ represents the regional fixed effect, which controls the factors that do not change with time at the regional level, and $\gamma_{t}$ represents the time fixed effect, which controls the characteristics that do not change with regional at the time level, and $\varepsilon_{it}$ is a random disturbance term.

### 3.2. Variable Selection

#### 3.2.1. Explained Variables

CO_2_ emissions: this paper refers to the measurement method of CO_2_ emissions in various regions by IPCC [43] and Wang [44] and calculates the CO_2_ emissions of eight primary energy sources: coal, coke, crude oil, gasoline, kerosene, diesel, fuel oil, and natural gas. The calculation formula is $CO_{2}=\sum_{1}^{8}E_{i}\cdot \xi_{i}\cdot \psi_{i}$, where *E_i_* represents the physical consumption of the i-th energy. The conversion coefficient ($\xi_{i}$) and carbon emission coefficient ($\psi_{i}$) of each fossil energy are shown in Table 1.

SO_2_: This paper uses annual provincial SO_2_ emissions to characterize this indicator and process it logarithmically.

PM_2.5_: Referring to the measure of PM_2.5_ by Shao et al. [45], this paper obtains the mean value of annual PM_2.5_ at the provincial level by parsing the satellite detection data and processing it logarithmically.

#### 3.2.2. Control Variables

Referring to the literature [35,36,46,47], and considering the homology of CO_2_, SO_2_, and PM_2.5_, this paper chooses a set of control variables. Mainly include:

(1) The real GDP per capita (lnPGDP). Based on the IPAT model [48], this paper takes real GDP per capita as the control variable. The GDP per capita is converted into real GDP per capita at constant prices in 2007 and then logarithmized to represent the economic development level.
(2) Energy intensity (ENIN). Energy intensity measures the energy utilization in production activities, and the higher the energy intensity, the more CO_2_ and air pollutant emissions are brought [36]. The energy intensity is expressed as a ratio of energy use to GDP.
(3) The proportion of secondary industry (INDU2). As the primary source of CO_2_ and air pollutant emissions, the higher the share of the secondary sector in economic development, the more serious the CO_2_ and air pollutant emissions [36]. The proportion of the secondary industry is expressed by the ratio of industrial economic added value to regional GDP and represents the characteristics of the overall industrial structure.
(4) Investment in fixed assets (lnINVE). Fixed assets investment projects, especially some high energy consumption and high emission projects, will increase carbon emissions and air pollution. The fixed assets investment price index is used to convert into fixed assets investment to real fixed assets investment in constant 2007 prices and then takes a logarithmic representation of it, representing economic activity.
(5) Social commodity retail (STRU). Referring to the practices of Wu et al. [49], the retail of social goods is included in the control variable. Expressed by the social commodity retail sales ratio to GDP represents the economic structure.
(6) Foreign direct investment (FDI). When studying the influencing factors of CO_2_ and air pollutant emissions, foreign direct investment’s impact on the environment needs to be considered. Foreign direct investment can bring advanced technology and promote energy conservation and emission reduction, but it may also bring environmental pollution [50,51]. It is expressed by the ratio of foreign direct investment to GDP.

#### 3.2.3. Mediating Variables

The following variables are selected as mediating variables to analyze the mechanism of ETS to achieve synergistic emission reduction, mainly including energy structure (ENST), measured by the ratio of coal consumption to total energy consumption [46]; technological progress (TECH), expressed by R&D investment intensity [41].

### 3.3. Data Sources and Descriptive Statistics

Because of the data availability, the data used in this paper are the balanced panel data of 30 provincial regions in China from 2007 to 2019, except Hong Kong, Macao, Taiwan, and Tibet. Among them, SO_2_ emissions are from the “*China Environmental Statistical Yearbook*”; coal consumption and energy consumption are from the “*China Energy Statistical Yearbook*”; R&D investment intensity is from the “*Statistical Bulletin of National Science and Technology Investment*” and relevant data of other variables are from the “*China Statistical Yearbook*”. “The descriptive statistics of the above-explained variables, control variables, and mediating variables are shown in Table 2.

## 4. Empirical Results and Hypothesis Testing

This section first reports the empirical results of DID model, that is, the impacts of ETS on CO_2_, PM_2.5_, and SO_2_ emissions, and then carry out a series of hypothesis tests.

### 4.1. Empirical Results

We first regress the model (1) using the two-way fixed effects model. The main results are shown in Table 3, columns (1) (3) (5) show the effects of ETS implementation on CO_2_, SO_2_, and PM_2.5_ when no control variables are added, and columns (2) (4) (6) show the effects when control variables are added. It is found that all regression results are significantly negative whether control variables are added or not, indicating that the implementation of ETS can indeed effectively reduce CO_2_ emissions and synergistically reduce SO_2_ and PM_2.5_ emissions, among which the impact on SO_2_ emission reduction is the largest, followed by CO_2_ and PM_2.5_.This paper uses columns (2) (4) (6) to explain and find that compared with the control group, the ETS reduces the emissions of CO_2_, SO_2_, and PM_2.5_ by 19.2%, 60.9%, and 6.4%, respectively. Hypothesis 1 is confirmed. This result is generally consistent with the views of the existing literature, which affirms the positive role of ETS. That is, ETS is beneficial for reducing carbon dioxide and air pollutant emissions in pilot areas and industries [52]. Moreover, it can be seen that real GDP per capita has a significant positive effect on SO_2_ and PM_2.5_ emissions, and energy intensity (ENEI) has a significant positive effect on CO_2_, SO_2_, and PM_2.5_ emissions. It shows that China is a high energy consumption country, and the increase in GDP per capita will lead to an increase in SO_2_ and PM_2.5_ emissions, and China should improve the energy efficiency [47,48,49,50,51,52,53].

### 4.2. Parallel Trend Test

The compelling premise of DID model is to meet the parallel trend assumption; that is, before the implementation of ETS, the CO_2_, SO_2_, and PM_2.5_ emissions of the pilot and non-pilot provinces have the same trend. Since the implementation of the ETS is not a comprehensive implementation at once, Beijing, Shanghai, Tianjin, Chongqing, Hubei, Guangdong, and Shenzhen are piloted from June 2013 to June 2014, so this paper takes 2014 as the pilot period of the above provinces. Fujian Province is piloted from 2017, so the status of a city in the experimental group or the control group will change. For example, Fujian Province was a non-pilot province in 2015, but it was a pilot province in 2017. Therefore, compared with the pilot and non-pilot provinces to draw a standard trend chart respectively [54], this paper draws lessons from Moser and Voena [55], uses the event analysis method, and constructs the following model to test its parallel trends.

(2) $$Emission_{it}=\beta_{0}+\sum_{k=-7}^{5}\beta_{k}Policy_{i,t-k}+\beta_{2}X_{it}+\alpha_{i}+\gamma_{i}+\varepsilon_{it}$$

where $Policy_{i,t-k}$ is a dummy variable. If region *i* has implemented the ETS in period *t-k*, then, $Policy_{i,t-k}=1$, otherwise the value is 0. The data in this paper is from 2007 to 2019, so it covers seven years before the implementation of the policy and five years after the implementation of the policy.

**Figure 2:**
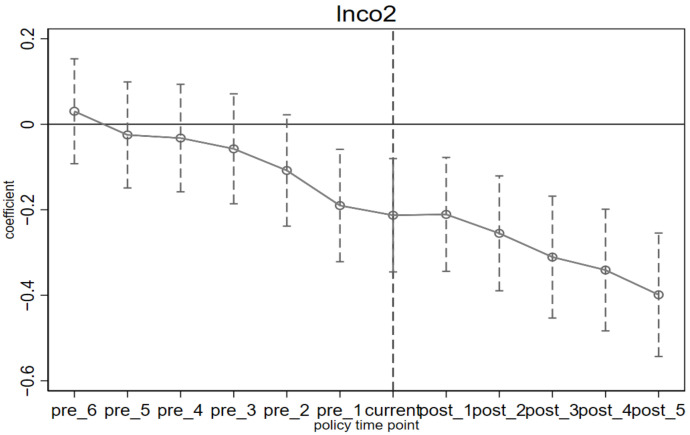
The difference in CO_2_ emissions before and after the implementation of ETS.

**Figure 3:**
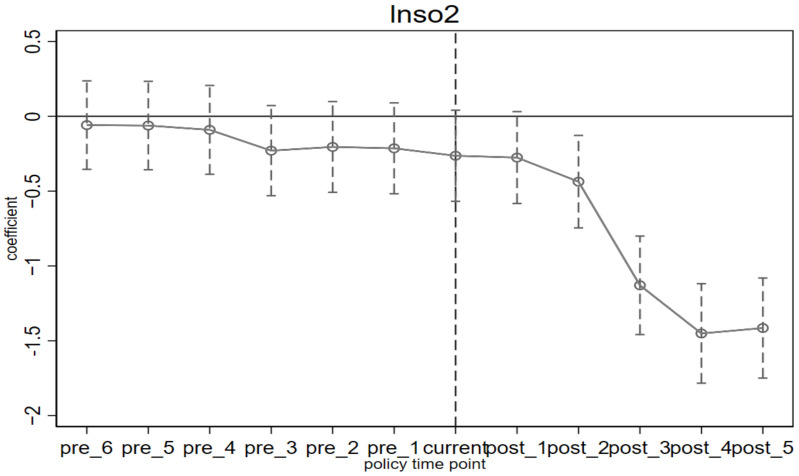
The difference in SO_2_ emissions before and after the implementation of ETS.

**Figure 4:**
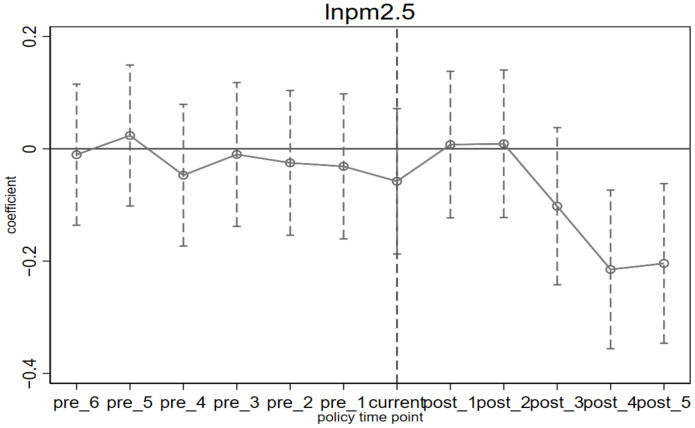
The difference in PM_2.5_ emissions before and after the implementation of ETS; Note: The small circle in the above figures represents the estimated coefficient *β_k_* obtained from Equation (2), and the dotted line is the 95% upper and lower confidence interval of *β_k_*. “pre” is before the policy, and “current” is the current period, and “post” is after the policy is implemented. (The same below).

To avoid the problem of multicollinearity, this paper discards the first period before the implementation of the policy, that is, 2007 as the base period. *β_k_* represents the difference in CO_2_, SO_2_, and PM_2.5_ emissions between pilot and non-pilot provinces in the *k* year after the implementation of the ETS. If *β_k_* has a relatively flat trend in the pre-period, with no significant increase or decrease, it is considered in line with the parallel trend construction. Otherwise, it is considered that there is already a significant difference between the pilot provinces and non-pilot provinces before the implementation of the policy, and the results of DID estimation are biased. The results of the parallel trend test are shown in Figure 2, Figure 3 and Figure 4, from which we can see that before the implementation of the policy, the estimated value of *β_k_* is very flat, indicating that there is no significant difference between the pilot provinces and non-pilot provinces before the implementation of the policy. From the perspective of dynamic effects, in Figure 2, in the year before the implementation of the ETS, although the ETS was not fully implemented, some pilot provinces and cities had already prepared for carbon trading activities in advance, so *β_k_* has been significant and consistently negative since 2013, which means that the effect of ETS began one year before the implementation of the policy, and in terms of value, the effect of CO_2_ emission reduction shows an increasing trend year by year, which may be related to the promotion of carbon trading market construction. Many studies have found that the effect of the policy has begun to appear one year before its implementation [28]. However, in Figure 3 and Figure 4, the estimated value of *β_k_* is significantly negative in the first and third years after the implementation of ETS, which indicates a time lag in the synergistic emission reduction effect of the ETS on SO_2_ and PM_2.5_ [36]. According to the above theoretical analysis, on the one hand, the implementation of ETS will make enterprises reduce coal consumption to adjust the energy structure, while the primary sources of PM_2.5_ are coal burning, motor vehicle emissions, industrial production process emissions, and dust, etc. The emission sources are complex, so other factors easily affect emissions. On the other hand, in the long run, enterprises will focus on developing low-carbon technologies and reducing carbon emissions through technological progress. However, technical research has the characteristics of significant investment and a long cycle [56], and finally, it may have a certain lag in coordinated emission reduction.

### 4.3. Dynamic Effect Test

Given the inconsistency of the policy significance periods of CO_2_, SO_2_, and PM_2.5_ in the parallel trend, and the policy effect has a lag, to avoid the endogeneity problem, this paper introduces the first-order lag of the explained variable added to the explanatory variable and uses the dynamic panel to verify whether the DID coefficient is still significant. The results are shown in Table 4. It can be found that after adding the first-order lag of lnCO_2_, lnSO_2_, and lnPM_2.5_, the coefficients of the lagged period are significant. The coefficients of DID are significantly negative whether or not control variables are added, which proves that the emission reduction effect of ETS does exist, rather than relying on the improvement of the environment in the previous period. The conclusion of the primary regression is reliable.

### 4.4. Placebo Test

**Figure 5:**
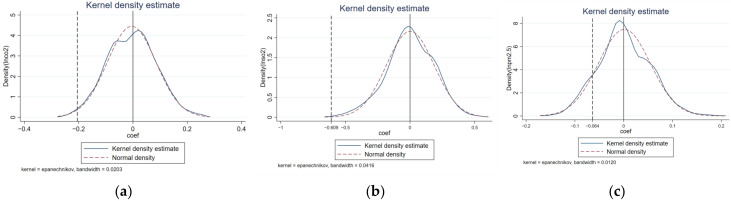
Subfigures (**a**–**c**) are the empirical cumulative distribution of placebo trial coefficients of CO_2_, SO_2_, and PM_2.5_, respectively. The solid line is the probability density distribution of the DID coefficients corresponding to the placebo test, the dashed line is the normal distribution, and the vertical dashed line indicates the estimated DID coefficients in columns (2) (4) (6) of Table 3.

This paper draws on Shi and Li [57] to conduct a placebo test by randomly selecting carbon emissions pilot cities. In this paper, seven provinces and cities are randomly selected from 30 provinces and cities as the fictitious experimental group and the others as the fictional control group. If the interaction term DID coefficient is not significant when the regression is based on the fictitious experimental group, the baseline regression results are robust. Considering that the policy implementation years are different in seven provinces and cities, this paper selects six provinces and cities to start the policy with one year in 2011–2015 and another province and city to start the policy with one year in 2016–2018, and repeats 1000 times, thus obtaining the DID regression estimated coefficients and probabilities for 1000 fictitious experimental groups and virtual policy time interactions. The obtained results are shown in Figure 5. As can be seen from the figure, the accurate DID estimated coefficients in columns (2) (4) (6) of Table 3 (−0.192, −0.609, and −0.064, respectively) are located in the low tails of the standard typical distribution plot, representing the truly estimated coefficients that are outliers in the estimated coefficients of the placebo test. Therefore, the conclusions of this paper can pass the placebo test, and the impact of ETS on CO_2_, SO_2_, and PM_2.5_ emissions in the pilot cities has little causal relationship with the omitted variables.

### 4.5. Random Grouping Test

The use of DID model requires the randomness of the implementation of ETS. Otherwise, it may cause the impact of sample selection on the results. This paper draws on Lu and Luo [58] for the analysis of CO_2_ emissions. Since the National Development and Reform Commission has determined the list of pilot cities at the end of October 2011, this paper analyzes the CO_2_ emissions of the pilot provinces and cities from 2007 to 2011, as shown in Table 5. It is found that before the pilot provinces and cities are determined, the lnCO_2_ emissions of the six provinces and cities are roughly in the middle level. This result shows that the implementation of ETS is not determined according to the amount of CO_2_ emissions. Therefore, it can be considered that the grouping of the treatment group and the control group in this paper satisfies the condition of randomness.

### 4.6. Exclude the Impact of Other Environmental Policies

In the process of estimating the impacts of ETS on CO_2_, SO_2_, and PM_2.5_ emissions, it may be interfered by the impact of other environmental policies in the same period, which will lead to deviations in the estimated effects of ETS. The “13th Five-Year Plan” mentioned that by 2020, the overall quality of the ecological environment would be improved, the green and low-carbon levels of production and lifestyle would rise, the total emissions of major pollutants would be significantly reduced, and the leading indicators of ecological environment protection, such as air quality and total emissions of pollutants, would be clearly defined. To accurately identify the emission reduction effect of the ETS, it is necessary to exclude the impact of the “13th Five-Year Plan”. This paper draws on the practice of [57], excluding the data from 2016 to 2019 for regression again. The regression results are presented in Table 6. The regression results show that the coefficient of “treat” is significantly negative at the 1–5% level, indicating that the conclusions of this paper are still robust after excluding other policy interference.

## 5. Analysis and Discussion

### 5.1. Mediating Effect Analysis and Discussion

The above results show that ETS can significantly reduce CO_2_ emissions and synergistically reduce PM_2.5_ and SO_2_ emissions. However, the mechanism of ETS affecting their emissions is still unclear, so this paper constructs the following mediating effect models.

(3) $$Emission_{it}=\alpha_{1}DID_{it}+\beta_{1}X_{it}+\mu_{i}+\nu_{t}+\varepsilon_{it}$$

(4) $$Mechan_{it}=\alpha_{2}DID_{it}+\beta_{2}X_{it}+\mu_{i}+\nu_{t}+\varepsilon_{it}$$

(5) $$Emission_{it}=\alpha_{3}DID_{it}+\alpha_{4}Mechan_{it}+\beta_{3}X_{it}+\mu_{i}+\nu_{t}+\varepsilon_{it}$$

where *Emission* includes CO_2_, SO_2_, and PM_2.5_, and *Mechan* is the mechanism variable. Based on the method proposed by Baron and Kenny [59], this paper tests the mechanism of ETS through the following four steps. First, according to model (3), the effect of ETS on CO_2_, SO_2_, and PM_2.5_ emissions is respectively tested. If the regression result *α*_1_ is significantly negative, it shows that ETS reduces CO_2_, SO_2_, and PM_2.5_ emissions, and *α*_1_ is the total effect of ETS. Second, according to model (4), regress the mediating variables with ETS dummy variables separately. If the regression coefficient *α*_2_ is significant, it indicates that the ETS has a significant effect on the intermediary variables. Third, according to model (5), the ETS dummy variables and mediating variables are put into the model at the same time to regress CO_2_, SO_2_, and PM_2.5_ emissions, respectively. Fourth, comparing the sign of *α*_3_ × *α*_4_ with *α*_2_, if the symbols are the same, and the absolute value of coefficient *α*_1_ is greater than that of *α*_3_, it indicates the existence of mediating effect, if the symbols are opposite, and the absolute value of *α*_1_ is less than that of *α*_3_, it indicates the existence of masking effect. This paper takes energy structure (ENST) and technological progress (TECH) as mediating variables.

First, this paper analyzes whether the ETS can reduce the emissions of CO_2_, SO_2,_ and PM_2.5_ by optimizing the energy structure. The specific test results are shown in Table 7. The first three columns remain unchanged, and column (4) is the regression result of the model (4). The coefficient of DID is significantly negative, indicating that the implementation of ETS has a significant negative impact on the energy consumption structure. According to the definition of the energy consumption structure in this paper, it can be seen that ETS can significantly reduce the proportion of coal consumption in the total energy consumption. That is to say; it can play a role in optimizing the energy consumption structure. After the implementation of the ETS, the actual carbon emissions of enterprises may be higher than the quota. At this time, it is necessary to purchase quotas from the carbon trading market to make up for the emission gap. In the long run, to reduce costs, enterprises will choose clean energy and reduce coal consumption, and then the energy structure is optimized. Columns (5) (6) (7) are the regression results of model (5), and the coefficients of ENST are all significantly positive. According to the definition of energy structure, it can be seen that energy structure optimization can reduce CO_2_, SO_2_, and PM_2.5_. The coefficients of DID and ENST are significant, and their symbol of the two coefficients is the same as that of the coefficient of ENST in column (4), and the absolute value of DID coefficient is smaller than that in columns (1) (2) (3) when ENST is included in the model, which indicates that the mediating effect of energy structure exists. That is to say, ETS can reduce the emissions of CO_2_, SO_2,_ and PM_2.5_ by optimizing the energy structure, and hypothesis 2 has been confirmed.

Then, this paper analyzes whether the ETS can reduce the emissions of CO_2_, SO_2_, and PM_2.5_ by promoting technological progress. The specific test results are shown in Table 8. The first three columns remain unchanged, and column (4) shows the regression results of model (4). The coefficient of DID is significantly positive, indicating that the implementation of ETS has a significant positive impact on technological progress. That is, it can promote technological progress. After implementing ETS, if the actual carbon emissions of enterprises are higher than the quota, they need to buy quotas from the carbon trading market to make up for the emission gap. In the long run, enterprises will carry out technological research and development to promote technological progress to reduce costs. Columns (5) (6) (7) are the regression results of model (5), and the coefficients of TECH are significantly negative, indicating that technological progress can significantly reduce the emissions of CO_2_, SO_2_, and PM_2.5_.The regression coefficients of DID and TECH are both significant, and the symbol of the product of the two coefficients is the same as that of TECH in column (4); the absolute values of the DID coefficient are smaller than that in columns (1) (2) (3) when TECH is included in the model, which indicates that the mediating effect of technological progress exists. Enterprises can use efficient production equipment and production processes, such as coal-fired unit desulfurization projects, sintering machine flue gas desulfurization projects, etc., thus reducing CO_2_, SO_2_, and PM_2.5_ emissions. That is, ETS can reduce the emissions of CO_2_, SO_2_, and PM_2.5_ by promoting technological progress. Hypothesis 3 is confirmed. ETS achieves technological progress and reduces carbon emissions, and enterprises can sell the surplus carbon quotas to gain revenue to cover the cost of pollution control and achieve economic welfare. This conclusion shows that the ETS gains economic benefits and realizes the Porter effect under the premise of clear property rights.

### 5.2. Heterogeneity Analysis and Discussion

The previous analysis shows that ETS can significantly reduce CO_2_ emissions and synergistically reduce SO_2_ and PM_2.5_ emissions in the pilot provinces and cities, so whether the emission reduction effect exists for regions with different levels of economic development and different provinces and cities. If so, whether there are differences in the reduction effect. To this end, the following analysis is conducted.

#### 5.2.1. Economic Heterogeneity

Referring to Zhang et al. [60], the sample provinces and cities are divided into economically developed and economically underdeveloped provinces and cities based on real GDP per capita. Then DID regressions are conducted separately. The results are shown in Table 9. The results show that the carbon emission reduction effect of ETS in developed provinces and cities is less than that of underdeveloped provinces and cities. ETS can reduce the CO_2_ emission of developed provinces and cities by 9.5%, while that of underdeveloped provinces and cities by 13.5%. Moreover, ETS has a significant synergistic effect on SO_2_ and PM_2.5_ emission reduction in underdeveloped provinces and cities, but not in developed provinces and cities. This may be because underdeveloped provinces and cities are in the primary stage of economic transformation, compared with developed provinces and cities, there will be more emissions of CO_2_ and air pollutants in their development process. The emission base of CO_2_ and air pollutants is significant, so the emission reduction benefit is relatively high. In addition, the energy intensity has a significant positive effect on CO_2_, SO_2_, and PM_2.5_ in underdeveloped provinces and cities, indicating that the underdeveloped provinces and cities still use conventional energy as the main driving force of economic development. Instead of realizing clean production, they rely on energy consumption, which aggravates air pollution, and the energy structure needs to be improved, with ample space for emission reduction.

#### 5.2.2. Regional Heterogeneity

Based on the baseline regression, the interaction term of each pilot province and city with DID was introduced in Equation (1) to analyze whether there is regional heterogeneity in the emission reduction effect of ETS. The results are shown in Table 10. It can be seen that (a) Beijing has the most potent carbon emission reduction effect, followed by Hubei and Shanghai, which is consistent with the conclusion drawn by Yi et al. [32]. Research shows that the emission reduction effect of ETS is mainly influenced by the coverage, allocation mode, and total amount setting and is positively related to the industry coverage of the carbon market. Beijing’s carbon market has a high intensity of administrative intervention and strict law enforcement, and it has included industries such as universities, medical institutions, and public transportation in its carbon market according to its industrial structure characteristics. Since its inception, the Shanghai carbon market has maintained 100% compliance for seven consecutive years. It has provided enterprises with energy-saving and emission reduction funds to encourage them to achieve green technological innovation actively. In Hubei province, 8% of the total carbon market quotas are reserved, and the reserved allowances indirectly increase the cost of carbon emissions for enterprises, which is conducive to improving the emission reduction effect. (b) Chongqing and Tianjin’s carbon emission reduction effect is at a medium level. At the same time, Guangdong is weak, which may be related to the continuous adjustment of Guangdong’s carbon quota auction policy, resulting in enterprises’ inability to judge the market trend and affecting the market activity correctly. (c) The ETS has played a significant role in synergistic emission reduction in the above carbon trading pilot areas. The synergistic emission reduction effect on SO_2_ is greater than that of PM_2.5_ because enterprises mainly achieve emission reduction through optimizing energy consumption structure and technological progress, while coal is the primary source of SO_2_ emission during energy consumption. The source and composition of PM_2.5_ are relatively complex. Regarding regions, Beijing and Shanghai, with higher carbon trading prices, have a more significant synergistic effect on SO_2_ emission reduction, probably because the higher the carbon price, the more significant the transaction cost, and the more aggressive enterprises will be in reducing emissions. In addition, the coordinated emission reduction will also be influenced by the trading volume, allocation method, and penalty intensity [35].

Overall, this paper empirically tests the impact of ETS on carbon emission reduction and synergistic emission reduction of air pollutants using a time-varying DID model based on panel data of 30 provinces from 2007–2019. To ensure that the pilot and non-pilot provinces and cities had the same development trend before the implementation of the policy, the parallel trend test was conducted first. Given that the significant policy periods of CO_2_, SO_2_, and PM_2.5_ in parallel trends are inconsistent and there are lags in the policy effects, a dynamic panel analysis is conducted by introducing first-order lags of the explanatory variables in this paper to avoid endogeneity problems. Then a series of robustness tests, such as placebo, were conducted to improve the accuracy of the benchmark regression. In addition, this paper also discusses the impact mechanism of ETS and the heterogeneity of ETS realization in different regions with different economic development levels and different pilot provinces and cities.

## 6. Conclusions

This paper takes ETS as the research object, divides the sample into pilot and non-pilot provinces and cities, and empirically investigates ETS’s synergistic emission reduction effect. The discussion on this issue can enrich the related research on synergistic emission reduction of ETS and realize the coordinated control of carbon dioxide and air pollutants in China. It can also provide a reference for other emerging developing countries that use ETS as an essential tool to achieve intended nationally determined contributions and have similar environmental problems.

The results show that (a) The implementation of ETS can significantly reduce CO_2_ emissions and synergistically reduce SO_2_ and PM_2.5_ emissions, and the synergistic effect on SO_2_ and PM_2.5_ emission reduction has a time lag. (b)The synergistic effect of ETS on SO_2_ emission reduction is higher than that of PM_2.5_. That is, ETS reduces air pollution mainly through synergistic SO_2_ emission reduction. (c) ETS mainly achieves the synergistic emission reduction of CO_2_ and air pollutants through two paths, one is to optimize the energy structure by controlling the total coal consumption and vigorously developing new energy, and the other is to promote technological progress by increasing R&D investment and low-carbon technologies promotion and application in source prevention, process emission reduction and, end-of-pipe treatment. (d) There is heterogeneity in the emission reduction effect of ETS for different economic development levels and different provinces and cities.

## 7. Policy Recommendations and Prospects

Based on the above research conclusions, the following policy recommendations are proposed. First, China should give play to the synergy of ETS and other environmental regulatory instruments. This paper finds that ETS has a synergistic effect on reducing air pollutants, so this policy will overlap with other regulatory instruments such as sulfur dioxide emissions trading. Therefore, in promoting the construction of the national carbon trading market, we should consider the appropriate setting of emission reduction targets and trading prices to realize further the synergistic emission reduction of carbon dioxide and air pollutants. Second, ETS can reduce emissions by optimizing the energy structure and promoting technological progress. Therefore, government departments should encourage technological innovation, reduce the proportion of coal consumption, promote the transformation of clean energy, accelerate the development of new energy, green environmental protection and other industries, and promote the development of the low-carbon economy. Third, China’s carbon trading market started late and has a short implementation year. Compared with developed countries, the trading mechanism is not perfect, and the trading efficiency is low. Although the ETS can significantly reduce carbon emissions, if we want to achieve the goal of carbon neutrality, carbon peak, its role must be further enhanced. The further promotion of the carbon market also needs to rely on effective administrative intervention by the government to achieve the compelling synergy between market incentives and administrative intervention. On the one hand, market instruments play a decisive role in ETS. The pilot areas should continue to strengthen the system construction of the carbon market, establish risk management and trading monitoring mechanisms, including verification and credit supervision, etc., to promote the coordination and linkage among various departments and industries. On the other hand, the government should play an influential regulatory role, strengthen trading supervision, improve the platform construction, formulate, and improve the legal system, make up for the problems of asymmetric information and poor transparency in the carbon market, and ensure the efficient operation of the carbon market.

To some extent, this study has enriched the research on the synergistic emission reduction effect of ETS, but there are still some shortcomings that need to be expanded. First, the synergistic emission reduction effect of ETS may be influenced by factors such as trading scale, trading price, allocation method, penalty intensity, etc. Based on this consideration, the synergistic reduction mechanism of ETS and its regional differences can be studied in the future, then more accurate regionally differentiated policy recommendations can be put forward. Second, ETS can bring economic, health, and social benefits in addition to emission reduction effects, and future research in this area can be carried out to enrich and improve the study of synergistic effects of carbon emission trading mechanisms.

## Figures and Tables

**Table 1:** Standard conversion coefficient and carbon emission coefficient of each energy.

Items	Coal	Coke	Crude Oil	Fuel Oil	Gasoline	Kerosene	Diesel	Natural Gas
$\xi_{i}$	0.71	0.97	1.43	1.43	1.47	1.47	1.46	1.33
$\psi_{i}$	0.76	0.86	0.55	0.59	0.59	0.57	0.62	0.45

**Table 2:** Descriptive statistics of variables.

Variable	Full Sample			Control Group			Treatment Group		
	Obs.	Mean	Std.D.	Obs.	Mean	Std.D.	Obs.	Mean	Std.D.
lnCO_2_	390	9.025	0.774	299	9.108	0.811	91	8.75	0.556
lnPM_2.5_	390	3.627	0.410	299	3.592	0.427	91	3.742	0.367
lnSO_2_	390	12.834	1.140	299	13.02	1.002	91	12.223	1.340
TECH	390	1.534	1.091	299	1.185	0.594	91	2.68	1.500
ENST	390	0.616	0.289	299	0.682	0.291	91	0.398	0.130
lnPGDP	390	10.379	0.524	299	10.232	0.445	91	10.862	0.472
lnINVE	390	9.036	0.904	299	9.016	0.969	91	9.1	0.647
ENIN	390	1.264	0.855	299	0.444	0.059	91	0.683	0.255
INDU2	390	0.428	0.082	299	0.435	0.074	91	0.406	0.103
STRU	390	0.393	0.071	299	0.389	0.075	91	0.408	0.049
FDI	390	0.022	0.017	299	0.018	0.015	91	0.033	0.019

**Table 3:** Benchmark regression results.

Variable	(1)	(2)	(3)	(4)	(5)	(6)
	lnCO_2_	lnCO_2_	lnSO_2_	lnSO_2_	lnPM_2.5_	lnPM_2.5_
DID	−0.181 ***	−0.192 ***	−0.638 **	−0.609 **	−0.078 **	−0.064 **
	(−4.84)	(−4.10)	(−2.73)	(−2.43)	(−2.47)	(−2.75)
lnPGDP		0.111		1.116 **		0.307 **
		(0.84)		(2.34)		(2.44)
lnINVE		0.092		0.246 **		–0.040
		(1.31)		(2.33)		(−1.46)
ENIN		0.090 ***		0.188 **		0.072 ***
		(4.97)		(2.77)		(8.96)
INDU2		0.307		0.328		−1.014 ***
		(0.97)		(0.35)		(−8.70)
STRU		−0.175		−0.886 *		−0.602 ***
		(−0.82)		(−1.79)		(−3.58)
FDI		−1.213*		−2.901		1.073*
		(−1.87)		(−1.24)		(2.15)
Constant	9.192 ***	2.925 ***	11.678 ***	−2.618	3.571 ***	1.172
	(1052.55)	(3.95)	(213.84)	(−0.53)	(483.06)	(0.91)
Obs.	390	390	390	390	390	390
City	YES	YES	YES	YES	YES	YES
Year	YES	YES	YES	YES	YES	YES
R-squared	0.575	0.604	0.814	0.832	0.227	0.311

*** *p* < 0.01, ** *p* < 0.05, * *p* < 0.10.

**Table 4:** Results of the dynamic effects test.

Variable	(1)	(2)	(3)	(4)	(5)	(6)
	lnCO_2_	lnCO_2_	lnSO_2_	lnSO_2_	lnPM_2.5_	lnPM_2.5_
L.lnCO_2_	0.807 ***	0.603 ***	0.681 ***	0.648 ***	0.598 ***	0.546 ***
	(109.47)	(10.31)	(5.13)	(4.73)	(6.08)	(4.42)
DID	−0.016 *	−0.030 *	−0.255 **	−0.262 *	−0.034 **	−0.030 **
	(−1.74)	(−1.89)	(−2.05)	(−1.77)	(−2.21)	(−2.33)
lnPGDP		0.293 ***		0.660		0.195 **
		(4.58)		(1.64)		(2.08)
lnINVE		−0.050 ***		0.112 *		−0.014
		(−3.78)		(1.90)		(−0.51)
ENIN		0.051 **		0.066		0.045 ***
		(2.46)		(1.28)		(3.55)
INDU2		1.218 ***		0.145		−0.552 ***
		(4.90)		(0.22)		(−4.61)
STRU		−0.018		−0.305		−0.150
		(−0.20)		(−0.80)		(−1.01)
FDI		−2.156 ***		−2.385		0.447
		(−2.85)		(−1.57)		(0.86)
Constant	1.777 ***	−1.135 *	3.548 **	−4.212	1.394 ***	−0.178
	(24.91)	(−1.95)	(2.24)	(−1.33)	(3.91)	(−0.15)
Obs	330	330	360	360	360	360

*** *p* < 0.01, ** *p* < 0.05, * *p* < 0.10.

**Table 5:** Comparison of CO_2_ emissions in carbon trading pilot provinces and cities.

Cities	2007	2008	2009	2010	2011
Beijing	6	5	4	4	3
Tianjin	8	7	8	9	8
Shanghai	15	15	14	14	13
Guangdong	24	23	23	23	23
Hubei	21	19	19	21	21
Chongqing	5	6	6	5	4

Note: the data in the table is the ranking of the CO_2_ emissions of pilot regions from small to large.

**Table 6:** Test for excluding interference from other policies.

Variable	(1)	(2)	(3)
	lnCO_2_	lnSO_2_	lnPM_2.5_
DID	−0.101 ***	−0.140 **	−0.062 ***
	(−4.77)	(−2.57)	(−3.98)
lnPGDP	0.244 ***	0.222 *	0.252 **
	(3.92)	(1.95)	(2.54)
lnINVE	0.308 ***	0.519 ***	−0.121
	(10.17)	(8.29)	(−1.61)
ENIN	0.118 ***	0.345 ***	0.059 **
	(8.04)	(9.91)	(2.87)
INDU	−0.303 ***	−1.091 ***	−1.160 ***
	(−4.78)	(−7.74)	(−7.48)
STRU	−0.438 *	−0.502 ***	−0.707 **
	(−2.06)	(−3.95)	(−3.33)
FDI	−1.061 ***	−0.533	1.452
	(−4.52)	(−0.66)	(1.72)
Constant	3.826 ***	6.152 ***	2.779
	(7.57)	(8.52)	(3.83)
Obs.	390	390	390
City	YES	YES	YES
Year	YES	YES	YES
R-squared	0.767	0.620	0.425

*** *p* < 0.01, ** *p* < 0.05, * *p* < 0.10.

**Table 7:** Test of mediating effect-energy structure.

Variable	(1)	(2)	(3)	(4)	(5)	(6)	(7)
	lnCO_2_	lnSO_2_	lnPM_2.5_	ENST	lnCO_2_	lnSO_2_	lnPM_2.5_
DID	−0.192 ***	−0.609 **	−0.064 **	−0.081 ***	−0.089 ***	−0.536 **	−0.054 **
	(−4.10)	(−2.43)	(−2.75)	(−3.95)	(−3.91)	(−2.30)	(−2.33)
ENST					1.267 ***	0.890 ***	0.122 *
					(22.52)	(4.36)	(1.84)
lnPGDP	0.111	1.116 **	0.307 **	−0.063	0.191 ***	1.172 *	0.315 **
	(0.84)	(2.34)	(2.44)	(−0.81)	(3.96)	(2.18)	(2.37)
lnINVE	0.092	0.246 **	−0.040	−0.039	0.142 ***	0.282 ***	−0.035
	(1.31)	(2.33)	(−1.46)	(−1.10)	(4.88)	(3.25)	(−1.16)
ENIN	0.090 ***	0.188 **	0.072 ***	−0.078 *	0.189 ***	0.257 ***	0.082 ***
	(4.97)	(2.77)	(8.96)	(−1.88)	(5.16)	(4.32)	(5.72)
INDU2	0.307	0.328	−1.014 ***	−0.110	0.446	0.426	−1.000 ***
	(0.97)	(0.35)	(−8.70)	(−1.09)	(1.72)	(0.47)	(−9.27)
STRU	−0.175	−0.886 *	−0.602 ***	−0.289 **	0.192	−0.629	−0.566 ***
	(−0.82)	(−1.79)	(−3.58)	(−2.34)	(1.61)	(−1.35)	(−3.33)
FDI	−1.213 *	−2.901	1.073 *	0.861 *	−2.305 ***	−3.667	0.968
	(−1.87)	(−1.24)	(2.15)	(2.07)	(−3.28)	(−1.45)	(1.69)
Constant	7.021 ***	−2.618	1.172	1.901 *	4.612 ***	−0.162	1.932
	(4.22)	(−0.53)	(0.91)	(1.85)	(10.46)	(−0.03)	(1.59)
Obs.	390	390	390	390	390	390	390
City	YES	YES	YES	YES	YES	YES	YES
Year	YES	YES	YES	YES	YES	YES	YES
R-squared	0.603	0.832	0.311	0.226	0.839	0.835	0.322

*** *p* < 0.01, ** *p* < 0.05, * *p* < 0.10.

**Table 8:** Test of mediating effect-technological progress.

Variable	(1)	(2)	(3)	(4)	(5)	(6)	(7)
	lnCO_2_	lnSO_2_	lnPM_2.5_	TECH	lnCO_2_	lnSO_2_	lnPM_2.5_
DID	−0.192 ***	−0.609 **	−0.064 **	0.206 **	−0.169 ***	−0.547 **	−0.045 **
	(−4.10)	(−2.43)	(−2.75)	(2.94)	(−4.35)	(−2.45)	(−2.85)
lnPGDP	0.111	1.116 **	0.307 **	−0.632 ***	0.039	0.926 *	0.248 **
	(0.84)	(2.34)	(2.44)	(−4.14)	(0.26)	(1.97)	(2.19)
lnINVE	0.092	0.246 **	−0.040	−0.044	0.087	0.233 **	−0.044
	(1.31)	(2.33)	(−1.46)	(−0.93)	(1.32)	(2.54)	(−1.46)
ENIN	0.090 ***	0.188 **	0.072 ***	0.113 ***	0.103 ***	0.222 ***	0.083 ***
	(4.97)	(2.77)	(8.96)	(4.79)	(5.99)	(3.14)	(11.01)
INDU2	0.307	0.328	−1.014 ***	0.811 ***	0.399	0.571	−0.939 ***
	(0.97)	(0.35)	(−8.70)	(4.67)	(1.22)	(0.60)	(−7.27)
STRU	−0.175	−0.886 *	−0.602 ***	1.225 **	−0.034	−0.519 *	−0.488 **
	(−0.82)	(−1.79)	(−3.58)	(3.02)	(−0.15)	(−1.94)	(−2.81)
FDI	−1.213 *	−2.901	1.073 *	−0.470	−1.267	−3.042	1.030 **
	(−1.87)	(−1.24)	(2.15)	(−0.55)	(−1.74)	(−1.17)	(2.30)
TECH					−0.115 **	−0.300 **	−0.093 **
					(−2.85)	(−2.66)	(−2.47)
Constant	7.021 ***	−2.618	1.172	8.185 ***	7.958 ***	−0.162	1.932
	(4.22)	(−0.53)	(0.91)	(4.80)	(4.35)	(−0.03)	(1.59)
Obs.	390	390	390	390	390	390	390
City	YES	YES	YES	YES	YES	YES	YES
Year	YES	YES	YES	YES	YES	YES	YES
R-squared	0.603	0.832	0.311	0.685	0.612	0.835	0.322

*** *p* < 0.01, ** *p* < 0.05, * *p* < 0.10.

**Table 9:** Economic heterogeneity analysis.

Variable	Developed Regions			Underdeveloped Regions		
	(1) lnCO_2_	(2) lnPM_2.5_	(3) lnSO_2_	(4) lnCO_2_	(5) lnPM_2.5_	(6) lnSO_2_
DID	−0.0944 ***	0.0472	−0.2980	−0.1096 ***	−0.0609 **	−0.1126 *
	(−3.743)	(1.273)	(−1.219)	(−3.389)	(−2.730)	(−1.940)
lnPGDP	1.9804 ***	0.5816 **	3.0065 ***	−0.7648 **	0.1574	−0.2525
	(10.387)	(2.295)	(4.679)	(−2.717)	(1.506)	(−1.006)
lnINVE	−0.0115	−0.0699	0.0280	0.0795	0.0183	0.4204 ***
	(−0.269)	(−1.138)	(0.122)	(0.868)	(0.365)	(6.475)
ENIN	0.7391 **	0.3925	0.8205	0.0966 ***	0.0806 ***	0.2889 ***
	(2.337)	(1.089)	(1.426)	(3.692)	(7.024)	(5.545)
INDU2	−2.5826 ***	1.5399 *	4.3789 ***	0.5304 *	−1.3904 ***	0.3011
	(−3.594)	(1.817)	(3.673)	(2.048)	(−5.253)	(0.295)
STRU	−0.5667 ***	−0.3809 *	−0.5116	0.2279	−0.6326 **	−0.8955
	(−5.240)	(−1.790)	(−1.015)	(0.705)	(−2.778)	(−1.309)
FDI	0.2181	0.3943	−8.9586 **	−0.3924	−0.0204	−0.3292
	(0.276)	(0.409)	(−2.212)	(−0.469)	(−0.010)	(−0.136)
Constant	12.0903 ***	−2.8836	24.4966 **	16.2044 ***	2.3510	10.6444 ***
	(−6.686)	(−1.008)	(−3.054)	(4.690)	(1.739)	(4.520)
Obs.	123	123	123	267	267	267
City	YES	YES	YES	YES	YES	YES
Year	YES	YES	YES	YES	YES	YES
R−squared	0.544	0.395	0.888	0.699	0.361	0.772

*** *p* < 0.01, ** *p* < 0.05, * *p* < 0.10.

**Table 10:** Regional heterogeneity analysis.

Variables	(1)	(2)	(3)
	lnCO_2_	lnSO_2_	lnPM_2.5_
DID	0.0264	0.1174	0.1966 ***
	(0.650)	(0.809)	(3.495)
DID × bj	−0.5862 **	−1.3716 **	−0.1715 *
	(−2.731)	(−2.408)	(−2.058)
DID × tj	−0.1655 ***	−0.8451 ***	−0.3880 ***
	(−5.103)	(−3.268)	(−3.116)
DID × sh	−0.2096 *	−1.3250 ***	−0.3080 **
	(−2.045)	(−3.379)	(−2.214)
DID × cq	−0.1941 ***	−0.5994 ***	−0.3477 ***
	(−3.093)	(−3.311)	(−5.140)
DID × hb	−0.2246 ***	−0.3713 **	−0.2845 ***
	(−5.679)	(−2.825)	(−4.195)
DID × gd	−0.1198 *	−0.4951 ***	−0.2289 ***
	(−2.101)	(−4.409)	(−4.007)
lnPGDP	−0.0490	0.5938 *	0.3927 ***
	(−0.210)	(2.052)	(3.086)
lnINVE	0.0470	0.1208	−0.0439
	(0.537)	(1.073)	(−1.134)
ENIN	0.0831 ***	0.1580 **	0.0666 ***
	(3.934)	(2.324)	(7.928)
INDU2	0.5833	0.3455	−1.2350 ***
	(1.441)	(0.354)	(−8.690)
STRU	−0.1276	−0.7906	−0.6730 ***
	(−0.501)	(−1.524)	(−3.232)
FDI	0.1944	−0.0821	0.8425
	(0.208)	(−0.048)	(1.180)
Constant	9.0403 ***	4.1606	0.3969
	(3.187)	(1.682)	(0.316)
Obs.	390	390	390
City	YES	YES	YES
Year	YES	YES	YES
R−squared	0.645	0.849	0.347

*** *p* < 0.01, ** *p* < 0.05, * *p* < 0.10.; Table 10 shows the regression results for the six pilot provinces and cities of Beijing (bj), Tianjin (tj), Shanghai (sh), Chongqing (cq), Hubei (hb), and Guangdong (gd) compared to the Fujian carbon market.

## Data Availability

Data are available from the authors upon reasonable request as the data need further use.
